# Histology of the heterostracan dermal skeleton: Insight into the origin of the vertebrate mineralised skeleton

**DOI:** 10.1002/jmor.20370

**Published:** 2015-03-30

**Authors:** Joseph N. Keating, Chloe L. Marquart, Philip C. J. Donoghue

**Affiliations:** ^1^ School of Earth Sciences, University of Bristol, Life Science Building24 Tyndall AvenueBristolBS8 1TQUK

**Keywords:** microstructure, jawless, dermoskeleton, gnathostome, bone, dentine, enameloid

## Abstract

Living vertebrates are divided into those that possess a fully formed and fully mineralised skeleton (gnathostomes) versus those that possess only unmineralised cartilaginous rudiments (cyclostomes). As such, extinct phylogenetic intermediates of these living lineages afford unique insights into the evolutionary assembly of the vertebrate mineralised skeleton and its canonical tissue types. Extinct jawless and jawed fishes assigned to the gnathostome stem evidence the piecemeal assembly of skeletal systems, revealing that the dermal skeleton is the earliest manifestation of a homologous mineralised skeleton. Yet the nature of the primitive dermal skeleton, itself, is poorly understood. This is principally because previous histological studies of early vertebrates lacked a phylogenetic framework required to derive evolutionary hypotheses. Nowhere is this more apparent than within Heterostraci, a diverse clade of primitive jawless vertebrates. To this end, we surveyed the dermal skeletal histology of heterostracans, inferred the plesiomorphic heterostracan skeleton and, through histological comparison to other skeletonising vertebrate clades, deduced the ancestral nature of the vertebrate dermal skeleton. Heterostracans primitively possess a four‐layered skeleton, comprising a superficial layer of odontodes composed of dentine and enameloid; a compact layer of acellular parallel‐fibred bone containing a network of vascular canals that supply the pulp canals (L1); a trabecular layer consisting of intersecting radial walls composed of acellular parallel‐fibred bone, showing osteon‐like development (L2); and a basal layer of isopedin (L3). A three layered skeleton, equivalent to the superficial layer L2 and L3 and composed of enameloid, dentine and acellular bone, is possessed by the ancestor of heterostracans + jawed vertebrates. We conclude that an osteogenic component is plesiomorphic with respect to the vertebrate dermal skeleton. Consequently, we interpret the dermal skeleton of denticles in chondrichthyans and jawless thelodonts as independently and secondarily simplified. J. Morphol. 276:657–680, 2015. © 2015 The Authors Journal of Morphology Published by Wiley Periodicals, Inc.

## INTRODUCTION

The vertebrate mineralised skeleton and its canonical cell and tissue types are among the most formative innovations in vertebrate evolutionary history. Unfortunately, their origins are poorly understood because living vertebrates either lack or possess all of the component mineralised skeletal systems in their entirety. The living jawless vertebrates, the cyclostomes (hagfishes and lampreys), possess only unmineralised cartilaginous fin radials, a braincase and rudiments of the vertebrae. In contrast, living jawed vertebrates possess mineralised axial, appendicular and dermal skeletons, a neurocranium and a splanchnocranium. Thus, there is a lack of experimental models representative of distinct grades in the evolutionary assembly of the vertebrate skeleton. However, there is a rich fossil record of jawless and jawed vertebrates, characterised as the ‘ostracoderms’, that record this episode (Donoghue and Purnell, 2005), revealing the gradual assembly of mineralised skeletal systems manifest in living jawed vertebrates (Donoghue and Sansom, 2002; Donoghue et al., 2006; Giles et al., 2013).

**Figure 1:**
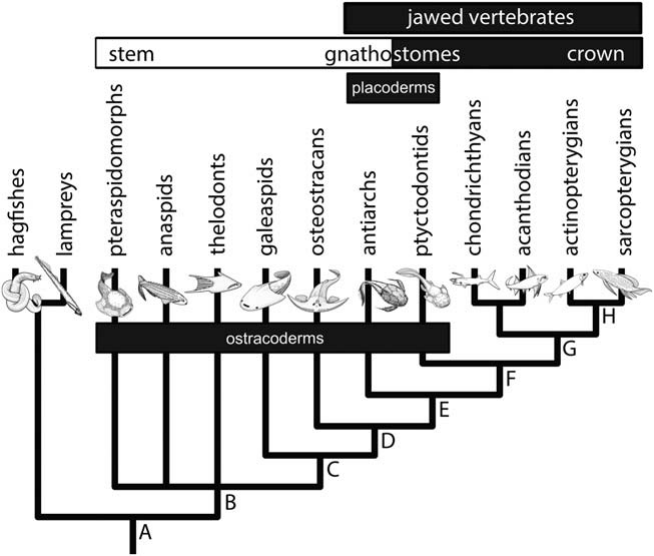
Phylogenetic relationships of the principal vertebrate groups from Donoghue and Keating (2014). Labelled internal branches refer to acquisition of key skeletal apomorphies. Origin of the vertebrate skeleton consisting of a splanchnocranium, neurocranium, fin rays and arcualia (**A**); origin of a mineralised dermal skeleton and of the canonical vertebrate mineralised tissues: bone, dentine and enameloid (**B**); origin of a mineralised neurocranium (**C**); cellular dermal and perichondral bone, neurocranium includes elements equivalent to the scapula and coracoid (**D**); mineralised splanchnocranium, evolution of jaws and pelvic girdle (**E**); mineralised axial skeleton (**F**), mineralised fin radials, teeth associated with spanchnocranium (**G**); and endochondral bone (**H**).

A mineralised dermal skeleton is manifest first in vertebrate evolutionary history (Fig. 1). It is in this skeletal system that the canonical skeletal cell and tissue types are apparent from the first (Donoghue et al., 2000; Donoghue and Sansom, 2002), with dermal bones comprising acellular bone surmounted by tooth‐like tubercles composed of enameloid, dentine and bone of attachment (Donoghue et al., 2006). The dermal skeleton comprises much of the skull table of fishes, along with their boney scales‐reduced in tetrapods to little more than the frontal bones of the face, palate and part of the shoulder girdle (Le Douarin and Kalcheim, 1999). The histological structure of these tissues is routinely preserved in exquisite detail and has been the subject of study for as long as the fossils have been known (e.g., Agassiz, 1835). However, despite these numerous studies, the nature of the primitive dermal skeleton remains unclear, principally because early attempts to synthesise these data were derived primarily from a strict stratigraphic reading of fossil occurrences (Halstead, 1969, 1973; Ørvig, 1967). More recently, cladistics has provided a framework in which to integrate these disparate data, yet current rudimentary knowledge of the diversity and evolution of the dermal skeleton in heterostracans precludes understanding the plesiomorphic vertebrate dermal skeleton. This is surprising, as heterostracans constitute one of the most diverse ostracoderm groups and they have been interpreted as the sister lineage to all other vertebrates with a homologous mineralised skeleton (Donoghue et al., 2000). Thus, although heterostracans do not directly evidence the plesiomorphic nature of the skeleton, through comparison to other skeletonising vertebrates, and within an explicit phylogenetic framework, it is possible to infer the plesiomorphic dermal skeleton. To that end, we survey the structure of the dermal skeleton across heterostracan diversity, resolve the plesiomorphic nature of the skeleton for heterostracans and, in drawing comparison to other skeletonising vertebrates, infer the nature of the primitive vertebrate dermal skeleton.

## HISTORICAL REVIEW

Agassiz (1835) provided the first descriptions of what we now recognise as the dermal skeletal plates of heterostracans from the Old Red Sandstone of Britain. While collectors had classified these fossils as molluscs or arthropods, Agassiz (1835, 1845) offered an alternative hypothesis: that these strange shells were the dermal skeletons of extinct fish. This hypothesis was contested by Kner (1847), who believed they were fossil mollusc shells, comparable to the ‘cuttlebone’ of *Sepia*. The matter was finally resolved in a classic study by Thomas Henry Huxley (1858). Huxley confirmed the vertebrate affinity of *Cyathaspis* and *Pteraspis* and was the first to attempt to characterise the heterostracan dermal skeleton. He discerned four discrete histological layers: a superficial layer of tubercle ridges composed of dentine, a compact layer of reticular canals circumscribed by thin lamellar walls, a cancellous layer of vertical lamellar walls enclosing polygonal chambers and a basal laminated layer. Following Huxley, Lindström (1895) described new material assigned to the genus *Cyathaspis* and revealed, for the first time, a highly organised linked vascular network comprising parallel superficial pulp canals, from which dentine canaliculi radiate, interlinked via a reticular network of canals, constituting the compact layer that opens into the polygonal chambers of the cancellar layer. These chambers interconnect via apertures perforating the lamellar walls, and open viscerally via occasional small canals perforating the basal layer.

Prior to these studies, the dermal skeleton of psammosteids had been described briefly by Agassiz (1835, 1845) and Pander (1857), yet this was overlooked by Lankester (1868), when he established the Heterostraci to unite several genera of fossil fish sharing similar histology. Thus, it was not until Traquair (1899) allied the psammosteids to the Heterostraci that comparison was drawn between the dermal skeleton of all these taxa. Traquair reasoned that psammosteids and other heterostracans share a common dermal skeletal architecture comprising a superficial layer of dentine tubercles or ridges, a vascular middle layer (comprising a homogenous layer of compact canals in psammosteids and differentiated into compact and cancellar layers in *Pteraspis* and *Cyathaspis*) and a laminated basal layer.

There have been numerous differing interpretations concerning the histology of the superficial layer of the heterostracan dermal skeleton. Pander (1857) first recognised the tubercles of the superficial layer comprise true dentine (i.e., perforated by fine calibre dentine tubules). He also noted the dentine tubercles are surmounted by a structureless layer, which he described as enamel‐like (Pander, 1857). Subsequent studies by Rohon (1893, 1902), Brotzen (1933) and Wills (1935) corroborated the presence of a discrete, homogenous, enamel‐like capping layer. Gross (1935), further noted the layer was optically distinct under polarised light, appearing as a bright band. As part of a study on the dermal skeleton of the Ordovician pteraspidomorph *Astraspis*, Bryant (1936) described comparable dentine tubercles also capped with a homogenous tissue, which he interpreted as a type of enamel. This interpretation was firmly rejected by Ørvig (1958), who observed that the seemingly homogenous capping layer in *Astraspis* is perforated by tubules, which he considered a characteristic of dentine. This was supported by Schmidt (1958), who termed the capping tissue ‘durodentine’. Halstead (Tarlo, 1960; Halstead Tarlo, 1964b) interpreted the crown tissue of heterostracans as a grade of dentine pervaded by terminal branches of dentine canaliculi. Later, Ørvig (1967) discriminated the capping layer of these early vertebrates as a discrete tissue in its own right, for which he considered the term ‘enameloid’ suitable. Ørvig (1967) reasoned that enameloid is developmentally distinct from both mammalian enamel and dentine in that it arises from a collagen rich extracellular matrix (ECM), which is subsequently reduced during hypermineralisation. In contrast, ‘true’ enamel develops from a noncollagenous ECM, produced by ameoblasts. Moss et al. (1964) demonstrated that collagen in the capping layer of sharks teeth and teleost scales is ectodermally derived, comparable to the enamel layer in tetrapod teeth. Consequently, he regarded these tissues as enamel, later differentiating them as ‘fiberous enamel’ (Moss, 1970). In accordance with this interpretation, he regarded the capping layer of heterostracans as enamel (Moss, 1968a). However, true enamel is genetically and developmentally distinct from ‘enameloid’ present in living teleosts or chondricthyans (Poole, 1967; Sasagawa, 2002; Kawasaki et al., 2004; Kawasaki and Weiss, 2006; Kawasaki et al., 2007) and is restricted to osteichthyans (Donoghue, 2001).

The growth of the superficial layer was elucidated by Gross (1961). He recognised that dentine lamellae were added centripetally about vascular cavities in a manner similar to the development of osteons in bone. Furthermore, Gross (1961) observed that the concentric dentine lamellae often grade imperceptibly and continuously into the concentric lamellae of the reticular layer, such that the two tissues can only be discriminated based on the presence/absence of a pervading fabric of canaliculi. In earlier studies, Rohon (1893, 1902) and Wills (1935) noted the superficial layer in some taxa is formed of discrete tubercle generations, interpreting buried tubercles as representing the most recent tubercle generation caught in the act of ‘erupting’ to the surface in much the same manner as teeth. Gross (1961), on the other hand, recognised successive generations of tubercles were accreted on top of, and overprinted, previous generations.

The middle layer of the heterostracan dermal skeleton was first characterised by Gross (1930) as part of his seminal study of psammosteid histology. Gross described the middle layer as comprising centripetal lamellar walls circumscribing large cancellous chambers. Under polarised light, he observed that lamellae appear as alternating light and dark bands, indicting that the crystal orientations and, by extension, the intrinsic collagen fibres of contiguous lamellae are perpendicular to each other. He also provided detailed description of a fabric of fine tubules, oriented orthogonally to the lamellae, which had previously been described by Gürich (1891) and Rohon (1893). Rohon (1893) speculated that these cavities may have housed simple bone cells without branching processes, but later rejected this view (Rohon, 1893, 1902). Gross (1930) regarded the tissue as acellular (i.e., lacking evidence of cell lacunae) and interpreted the cavities as unmineralised collagen fibre spaces, later specifically identifying them as Sharpey's fibres (Gross, 1935). Gross (1930) coined the lamellar tissue ‘aspidin’, and regarded it as neither a type of bone, nor a type of dentine, but a distinct mineralised tissue related to both bone and dentine. Obruchev (1941, 1964) later resurrected Rohon's (1893) hypothesis, interpreting the tissue as cellular and the thread‐like cavities as cell lacunae or canaliculi. This interpretation was supported by Halstead (1969, 1973, 1974; Halstead Tarlo, 1963, 1964a, 1964b, 1965) and Novitskaya (1966). The opposing view, that the elongate spaces represent voids of unmineralised collagen fibres (most frequently identified as Sharpey's fibres), was maintained by Gross (1935), Bystrow (1955), Ørvig (1951, 1965, 1967, 1968), and Moss (1968b). Meanwhile, although Denison (1967) seems to have supported this view, he also interpreted some of the spaces as having originally housed odontoblast cell‐processes (at least in *Astraspis*). Inevitably, confusion over the identity of the unmineralised aspidin spaces spilled into the wider debate concerning the primitiveness of cellular versus acellular bone. Within this context, aspidin has variously been interpreted as a primitive acellular type of bone (Denison, 1963; Halstead, 1987), a secondarily acellular bone (Stensiö, 1927; Ørvig, 1951, 1965), a primitive type of cellular bone (Obruchev, 1941, 1964; Halstead Tarlo, 1963, 1964a, 1964b; Halstead, 1969), a type of dentine (Urist, 1962, 1964a, 1964b; Denison, 1967) and an intermediate grade between dentine and bone (Halstead, 1973). Urist (1962, 1964a, 1964b) argued that aspidin could not be a type of bone on the grounds that it did not undergo remodelling, yet tentative evidence of resorption was reported in the psammosteids in the form of scalloping of concentric aspidin lamellae (Gross, 1930; Halstead Tarlo, 1963, 1964a, 1964b).

The lamellar tissue of the basal layer was included by Gross (1930) in his original definition of aspidin and later differentiated by Halstead (1969) as ‘lamellar aspidin’, based on the presence of obliquely aligned true Sharpey's fibres. Wang et al. (2005) and Donoghue et al. (2006) drew comparison between the basal plywood‐like layer in heterostracans and osteostracans and the basal ‘isopedin’ layer of the osteichthyan dermal skeleton. The growth of this tissue layer was first considered by Stensiö (1927). Stensiö observed ‘delicate striations’ within individual laminae of the basal layer of pteraspids, which he interpreted as evidence of intrinsic fibres. He noted that the striations of contiguous lamellae were orientated perpendicular to each other. Following this line of evidence, he suggested that development of the heterostracan dermal skeleton was achieved in a similar manner to the development of the corium aponeurosis, as outlined by Holmgren (1920), in which successive fibroblast strata form at the base of the dermis and give rise to orthogonal layers of collagen fibres. Stensiö (1927) believed that, while there was no evidence of cell spaces in the heterostracan dermal skeleton, cells akin to fibroblasts must have been present during ossification, residing between laminae. The basal apposition of lamellae was corroborated by Gross (1935), Fahlbusch (1957), Denison (1973) and White (1973).

Such a wealth of histological studies evidences the perceived importance of these fossils within the context of early vertebrate evolution. Resolving the nature of the primitive heterostracan dermal skeleton will yield fundamental insight into the ancestral skeleton shared by all skeletonising vertebrates, ourselves included. Yet it is the inconsistencies between these different histological accounts that have hampered attempts to resolve the plesiomorphic heterostracan skeletal bauplan. On the one hand, it appears different taxa exhibit markedly different adult dermal skeletal architectures. For example, in some heterostracans, the superficial layer shows evidence of addition of tubercles (Gross, 1961), while in others it does not (e.g., Denison, 1973; Halstead, 1973). In some taxa, the middle layer can be subdivided into two histologically distinct layers (Lindström, 1895; Kiaer, 1932), whereas in others it appears homogenous (Gross, 1930). On the other hand, differing interpretations regarding the microstructure of discrete tissues have confused, rather than informed, understanding of heterostracan histology. For example, the published literature is equivocal on the presence of enameloid (Ørvig, 1967 vs. Halstead, 1973) and whether the middle layer is a type of bone (Ørvig, 1951; Halstead, 1987), dentine (Urist, 1962; Denison, 1967) or an intermediate tissue grade (Halstead, 1973).

## MATERIALS AND METHODS

We set out to survey the diversity of heterostracan skeletons, recharacterize the dermal skeletal histology for each major heterostracan lineage and, with reference to previous palaeohistological studies, infer the plesiomorphic nature of the vertebrate skeleton. We sampled eight heterostracan taxa encompassing the phylogenetic diversity of the Heterostraci *sensu* Janvier (1996). Each major heterostracan lineage (including amphiaspids, cyathaspids, pteraspids, psammosteids, corvaspids, tranquairaspids) is represented by at least one taxon. Additionally, we analysed the dermal skeletal histology of two tessellate heterostracans, namely *Tesseraspis* and *Lepidaspis*. We analysed the histology of the cephalothoracic shield in each lineage and, where material was available, we also studied the histology of the body squamation. Data were collected using scanning electron microscope (SEM) Back‐Scattered Electron (BSE) analysis of polished sections, optical microscopy of thin sections and Synchrotron Radiation X‐ray Tomographic Microscopy (SRXTM) of scales and plate fragments.

The material of this study originates from a number of geological localities. *Lepidaspis serrata* Dineley & Loeffler and *Pteraspis* sp. material were collected from the Drake Bay Formation of Prince of Wales Island in the Canadian Arctic Archipelago and is stored at the Naturhistoriska Riksmuseet, Stockholm (NRM‐PAL C.4940‐45). The *Tesseraspis tesselata* Wills, *Phialaspis symondsi* Lankester, *Corvaspis kingi* Woodward and *Anglaspis macculloughi* Woodward material were collected from a small stream section at Earnstrey Hall Farm, Shropshire, UK (see Wills, 1935; Ball et al., 1961) and is stored at the Natural History Museum, London (NHM P.73614‐21). *Loricopteraspis dairydinglensis* White is from a stream section in nearby Dairy Dingle (see Ball et al., 1961) and is also stored at the NHM (NHM P.73622‐23). The *Amphiaspis* sp. was recovered from a borehole in the Norilsk Region of Siberia and is stored at the Institute of Geology, Tallinn University of Technology (GIT 313‐32). The *Psammosteus megalopteryx* Trautschold specimen was received from the collection of the late Beverly Halstead and is of unknown provenance. It has been accessioned at the NHM (NHM P.73624).

### Scanning Electron Microscopy

Specimens were embedded using Struers EpoFix® resin and left to cure for at least 24 h. Specimens were cured in a vacuum chamber for the first 30 min, in order to remove excess gas and reduce bubble formation. Sections were cut using a Buehler IsoMet® low speed saw. Cut surfaces were then impregnated using Buehler EPO‐THIN® resin. Smaller specimens were mounted within 25 mm diameter aluminium rings using Buehler EpoFix®. Specimens were manually ground using grit sizes ranging from P1200 to P4000. Buehler IsoCut® Fluid for lubrication was used during all grinding and polishing. Large specimens were polished manually using Buehler MetaDi® 6 and 1 μm Diamond Paste. Smaller specimens mounted within aluminium rings were instead polished using a Buehler Ecomet® 250 Grinder–Polisher using Buehler MetaDi® 3 and 1 μm suspensions. Polished samples were carbon coated using a Emitech K450 carbon coater. The specimens were imaged using a Hitachi S‐3500N SEM. This work was undertaken at the University of Bristol School of Earth Science's Electron Microbeam Facility.

### Synchrotron Radiation X‐ray Tomographic Microscopy

SRXTM experiments were conducted at the TOMCAT beamline of the Swiss Light Source, Paul‐Scherrer Institut, Villigen, Switzerland. Measurements were taken using 10× and 20× objective lenses, exposure times of 100–200 ms and energies of between 13 and 18 KeV. Thousand five hundred and one equiangular projections were acquired over 180° for each dataset. Projections were postprocessed and rearranged into flat‐ and dark‐field‐corrected sinograms, and reconstruction was performed on a 60‐core Linux PC farm, using a highly optimised routine based on the Fourier transform method and a regridding procedure (Marone and Stampanoni, 2012) resulting in volumetric data with voxel dimensions of 0.65 and 0.325 μm, respectively. These data were analysed, manipulated and segmented digitally using AVISO 8.0 standard edition software for computed tomography developed by Visualisation Sciences Group. Tomographic sections were produced using the orthoslice module. Virtual thin sections were produced using the volume‐rendering module and cropping the data, producing virtual sections 10–100 slices thick. Three‐dimensional virtual models of scales and plates were generated using the isosurface module.

### Light Microscopy

Thin sections were examined at the University of Bristol using a Leica M250C microscope with a 2× Plan Apochromat lens and imaged with a Leica DFC425C digital camera.

## RESULTS

### Tesselate Heterostracans

#### Lepidaspis serrata

**Figure 2:**
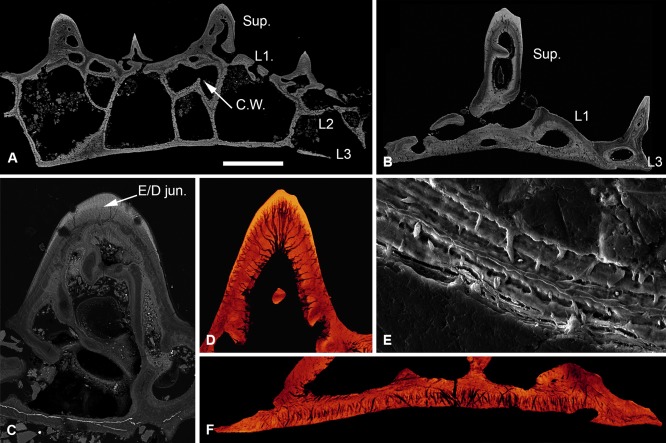
Histology of *Loricopteraspis serrata*. NRM‐PAL C.5939, SEM BSE section through the cephalothoracic shield (**A**); NRM‐PAL C.5940, SRXTM section through a scale unit (**B**); NRM‐PAL C.5941, SEM BSE section through a tubercle, showing the enemeloid capping layer (**C**); NRM‐PAL C.5940, SRXTM volume rendered virtual thin section of a tubercle, showing the arrangement of dentine canaliculi radiating from a pulp canal (**D**); NRM‐PAL C.5942, etched SEM section through a wall of L2, showing homogenous core and lamellar margins pervaded by an orthogonal fabric of thread‐like spaces (**E**); NRM‐PAL C.5940, SRXTM volume rendered virtual thin section of L3, showing Sharpey's fibres trending in two principal orientations (**F**). Sup., superficial layer; L1, layer 1; L2, layer 2; L3, layer 3; C.W., incomplete cross wall; E/D jun., Enameloid/dentine junction. Scale bar equals 606 μm in (A), 227 μm in (B), 179 μm in (C), 55 μm in (D), 30 μm in (E) and 56 μm in (F).

The dermal skeleton of *Lepidaspis* is composed of a broad cephalothoracic shield with body armour of separate scale units. The cephalothoracic shield is constructed from four distinct layers (Fig. 2A); a superficial layer of oak leaf‐shaped tubercles, a discontinuous compact layer of canals (L1), a cancellous layer (L2) and a thin basal layer (L3). The scale units equate to the superficial, compact and basal layer of the shield, but lack the middle cancellous division (Fig. 2B). The *Lepidaspis* dermal skeleton shows no evidence of cell lacunae within the mineral matrix.

##### Superficial layer

Composed of elongate discrete dentine tubercles capped with enameloid (Fig. 2C). Each tubercle contains a network of vascular canals, interpreted as pulp canals, about which dentine is developed centripetally, and from which highly polarised odontoblast canaliculi (usually 1–2 μm diameter) radiate. The canaliculi pass through the dentine and permeate the enameloid/dentine junction, at which point they ramify (Fig. 2D). There are no odontoblast lacunae. The enameloid is up to 50 μm thick and aside from the permeating ondontoblast canaliculi, is structureless, compatible with its interpretation as single crystallite enameloid (SCE) *sensu* Gillis and Donoghue (2007). The superficial layer of both the scales and the cephalothoracic shield is expanded peripherally by marginal accretion of new tubercles, producing lines of arrested growth.

##### Layer 1

A layer of compact canals, measuring approximately 200 μm thick, from which the pulp canals branch. The canals are circumscribed by osteon‐like centripetal lamellar walls, which contain a sparse orthogonal fabric of thread‐like spaces. In the scale units, the compact layer comprises a single continuous stratum of canals, which links the vasculature of the scale (Fig. 2B). In the cephalothoracic shield, L1 is discontinuous, occurring as a compact region at the base of each tubercle and opening into L2 (Fig. 2A).

##### Layer 2

Comprises large polygonal cancellae separated by thin walls measuring approximately 50 μm in diameter. The cancellae are interconnected via small canals penetrating the separating walls. Small canals also link the cancellae to the overlying superficial vascular network and open externally via pores between the tubercles. The histological construction of the middle layer is comparable regardless of the arrangement of the vascular network. The vascular cavities are enclosed by concentric lamellar tissue, which show evidence of centripetal apposition like osteons. The separating walls are pervaded by a coarse fabric of thread‐like spaces, around 2–10 μm in diameter, which are oriented orthogonal to, and cross cut, the lamellae (Fig. 2E). The core of the walls, on the other hand, is homogenous and shows no evidence of an unmineralised fabric. Occasionally, cancellae are partially subdivided by incomplete crosswalls (Fig. 2A), indicating subdivision of the vascular spaces occurred through ontogeny.

##### Layer 3

Extremely thin in both the scales and shield (less than 70 μm). It comprises an avascular plywood‐like tissue formed from around 3–5 ply. The basal layer is penetrated by a dense fabric of coarse unmineralised spaces, each approximately 2–5 μm in diameter. These spaces are orthogonal relative to the basal lamellae and trend in two principal orientations perpendicular to one another (Fig. 2F). Given their topological occurrence in the deepest layer of the dermal skeleton, these spaces are interpreted as voids that contained Sharpey's fibres.

#### Tesseraspis tesselata

**Figure 3:**
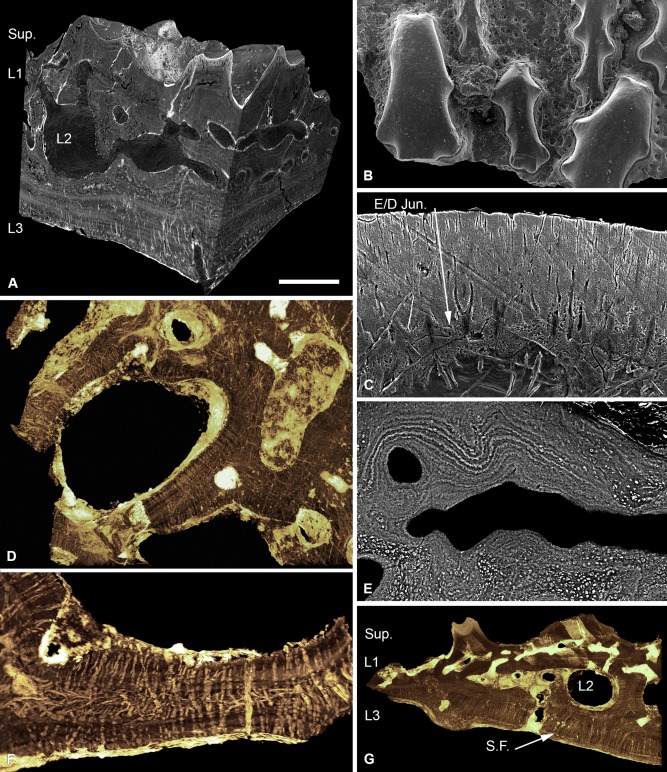
Histology of *Tesseraspis tesselata*. NHM P.73617, SRXTM sections through an isosurface model of a tessera (**A**); SEM of the external surface morphology of a tessera, showing two distinct tubercle generations, specimen lost (**B**); etched SEM section through the enameloid capping layer of the superficial layer, specimen lost (**C**); NHM P.73617, SRXTM volume rendered transverse section through L2, showing the architecture of the intersecting radial walls (**D**); NHM P.73618, SEM BSE section through L2 showing truncated centripetal lamellae interpreted as resorption (**E**); volume rendered virtual thin sections of NHM P.73617 (**F, G**); transverse section through a radial wall of L2, showing the arrangement of thread‐like spaces (F); section through the dermal skeleton of a tessera, showing the arrangement of Sharpey's fibres in L3 (G). S.F., Sharpey's fibres. Scale bar equals 193 μm in (A), 628 μm in (B), 47 μm in (C), 124 μm in (D), 68 μm in (E), 64 μm in (F) and 230 μm in (G).

The cephalothoracic shield of *Tesseraspis* is composed of numerous tessellating plates. These are four‐layered (Fig. 3A), consisting of a superficial layer of elongate ridges, a compact layer of canals (L1), a trabecular middle layer (L2) and a substantial lamellar basal layer (L3).

##### Superficial layer

Formed of at least two discrete tubercle generations (Fig. 3B). The first generation comprises elongate globular ridges, approximately 400 μm in width, resembling the oak‐leaf‐shaped tubercles of *Lepidaspis*. A second generation of similarly shaped, but larger, globular ridges, approximately 850 μm in width, are accreted obliquely on top of the first generation tubercles producing a discontinuity between successive generations. Both tubercle generations are composed of dentine, which develops centripetally about ascending pulp canals. A discontinuous, avascular layer of SCE, up to 100 μm thick, caps each tubercle. Polarised odontoblast canaliculi radiate from the pulp canals and perforate the dentine. At the enameloid/dentine junction, the canaliculi begin to ramify and their branches permeate the enameloid (Fig. 3C). The enameloid layer in some tubercles is discontinuous or even entirely absent, presumably due to extensive wear.

##### Layer 1

Measured approximately 300 μm in thickness. Ascending pulp canals of the superficial layer branch from a compact anastomosing network of canals, circumscribed by osteon‐like centripetal lamellar walls. The lamellar tissue is homogenous and appears to lack a pervading fabric of unmineralised spaces. The canals interlink with those of the middle layer (L2) and open externally via numerous pores between tubercles (Fig. 3B).

##### Layer 2

Measured approximately 1.1 mm in thickness. Constructed from a compact mesh of intersecting radial walls that define an anastomosing network of canals and occasional large chambers (Fig. 3D). The radial walls exhibit microstructural differentiation in section. The core of the radial walls is formed of an undifferentiated tissue, which is perforated by a dense mesh of concentrically arranged, discontinuous, coarse thread‐like spaces, each approximately 3–5 μm in diameter. The outer portions of the walls are constructed from concentric lamellae, which develop centripetally enclosing the vascular spaces. These concentric lamellae are frequently truncated by the vascular spaces, indicating that L2 was systematically remodelled during development (Fig. 3E). The centripetal tissue also contains a fabric of coarse thread‐like spaces, yet these are aligned orthogonal to the lamellae rather than concentrically enveloping the vascular space. Close to the boundary between the centripetal tissue and undifferentiated core, thread‐like spaces are observed to change orientation along their length. Thus, in section, the orthogonal discontinuous spaces appear to radiate either side of a concentric mesh of spaces enveloping the vasculature (Fig. 3F). The thread‐like spaces are more prevalent in the lower portion of L2.

##### Layer 3

Measured up to 400 μm in thickness and is relatively well developed in comparison to other heterostracan taxa. It is composed of 8–12 discrete lamellae, which are perforated by an orthogonal fabric of discontinuous, coarse, parallel extrinsic fibres (Fig. 3G). These measured around 3–9 μm in diameter and are interpreted as Sharpey's fibres. Ascending canals of the linked vascular network sporadically penetrate L3.

### Corvaspidiformes Stensiö, 1964

#### Corvaspis kingi

**Figure 4:**
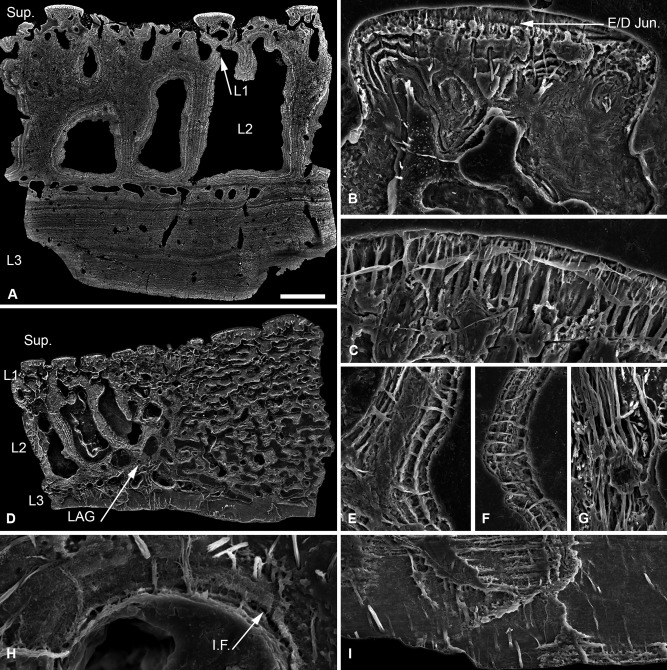
Scanning electron microscopy BSE (**A**) and etched SEM sections (**B–I**) through the cephalothoracic dermal skeleton of *Corvaspis kingi*. NHM P.73613, Sectioned fragment from the centre of the headshield (A); NHM P.73614, superficial tubercle, showing centripetal dentine lamellae (B); NHM P.73614, enameloid capping layer, showing fine calibre ramifying canaliculi (C); NHM P.73615, section through the margin of the shield, showing the transition from cancellar to trabecular L2 (D); sections through radial walls of L2 (E–G), showing thread‐like spaces radiating from a homogenous core, NHM P.73614 (E), thread‐like spaces pervading the entire thickness of the wall, NHM P.73615 (F), and a region of linearly arranged thread‐like spaces, NHM P.73616 (G); NHM P.73614, L2 osteon, showing an intrinsic parallel fibred matrix (H); NHM P.73614, L3 showing an orthogonal fabric of Sharpey's fibres (I). LAG, line of arrested growth; I.F., intrinsic fibres. Scale bar equals 385 μm in (A), 44 μm in (B), 35 μm in (C), 569 μm in (D), 40 μm in (E), 55 μm in (F), 42 μm in (G), 16 μm in (H) and 62 μm in (I).

The dermal skeleton of *Corvaspis* is composed of a superficial layer of tubercles and elongate ridges overlying a compact layer of canals (L1). The middle layer of the shield (L2) is cancellous with trabecular margins, while the basal layer (L3) is lamellar (Fig. 4A).

##### Superficial layer

Arrangement of tubercles gives the appearance that the shield is formed of discrete units. The tubercles themselves are composed predominantly of dentine capped with a layer of SCE up to 16 μm thick. They contain one or several ascending pulp canals, about which dentine develops centripetally and from which poloarised odontoblast canaliculi (approximately 4 μm in diameter) radiate (Fig. 4B). The canaliculi permeate the dentine and ramify at the enameloid/dentine junction. The terminal branches pervade the enameloid layer (Fig. 4C).

##### Layer 1

Measured approximately 200–300 μm in thickness. The pulp canals of the superficial layer branch from a compact stratum of anastomosing canals, circumscribed by osteon‐like centripetal lamellar walls. The lamellar tissue is homogenous and lacks a conspicuous pervading fabric of unmineralised spaces. In the centre of the shield, the compact canal network forms a well‐defined layer. However, in the trabecular marginal region, the canals of L1 pass imperceptibly into L2 (Fig. 4D).

##### Layer 2

Measured approximately 1.5–2 mm in thickness. L2 comprises a vascular network circumscribed by intersecting radial walls. The centre (or core) of the shield consists of polygonal cancellae, while the peripheral margins and base of the layer is trabecular (Fig. 4D). The intersecting radial walls, which define the vascular network, are composed of centripetal lamellar tissue, which shows osteon‐like development about the vascular spaces. In some sections, the walls are differentiated into outer lamellar and central homogenous layers (Fig. 4E). Two distinct fabrics permeate the intersecting walls. The most conspicuous fabric is composed of fairly coarse thread‐like spaces, each approximately 3–6 μm in diameter. Typically, these thread‐like spaces are aligned orthogonal to the concentric lamellae (Fig. 4E,F), although occasionally they form a dense linear fabric (Fig. 4G). In sections where the wall exhibits a ubiquitous lamellar construction, the thread‐like spaces cross cut the entire width of the walls (approximately 50–100 μm thick; Fig. 4F). However, in sections where the walls are differentiated, the thread‐like spaces appear to radiate from the undifferentiated centre (Fig. 4E), suggesting that this layer develops from remodelling of lamellar tissue following or during formation of the thread‐like spaces. The second fabric consists of minute, intrinsic, discontinuous fibrils, which span single lamellae and measure less than 1 μm in diameter (Fig. 4H). The intrinsic fibrils of each consecutive lamella share a similar orientation orthogonal to the centripetal tissue. Consequently, the tissue is best interpreted as parallel fibred bone (PFB) *sensu* De Ricqlès et al. (1991). The trabecular region of L2, which envelops the cancellous core, is interpreted as a late growth stage, which has accreted onto the margin of the shield. A line of arrested growth defines the boundary between the marginal trabecular and central cancellous divisions. This line is obscured in places by trabecular osteons, which develop via remodelling (Fig. 4D).

##### Layer 3

Extremely thick (up to 800 μm) and is composed of over 40 ply. It is avascular, save for occasional ascending canals opening into the vascular network of L2. It contains a conspicuous coarse fabric of parallel extrinsic fibres, which are oblique—orthogonal in relation to the lamellae (Fig. 4I). Each fibre measured up to 5–10 μm in diameter. They are compatible with Sharpey's fibres.

### Traquairaspidiformes Tarlo, 1962

#### Phialaspis symondsi

**Figure 5:**
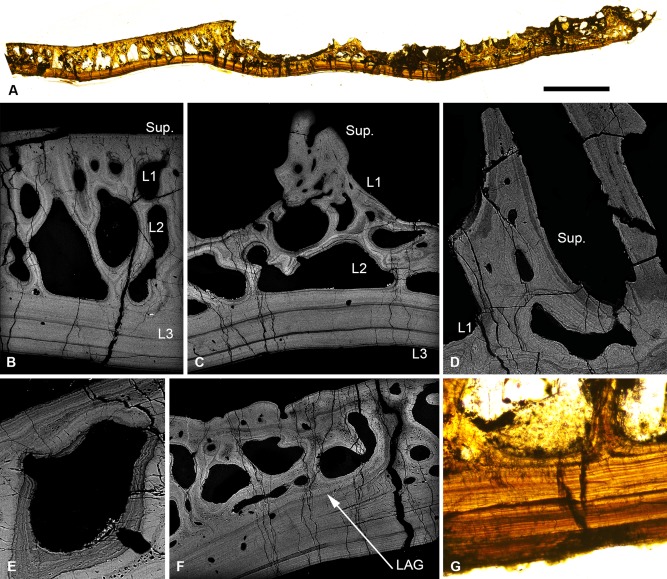
Light microscopy thin section (**A, G**) and SEM BSE polished sections (**B–F**) through the cephalothoracic shield of *Phialaspis symondsi*, NHM P.73619. Section from the centre to the margin of the shield, showing the transition from the central ‘plate’ to the peripheral tuberculated region (A); histological structure of the central ‘plate’ (B) and peripheral region (C); detail of the superficial layer/L1 of the peripheral region. L1 centripetal lamellae are truncated, suggesting the vascular canals were remodelled via resorption (D); osteon lamellae of L2 truncated by vascular space, indicating resorption (E); section through the peripheral region, showing line of arrested growth demarking the previous margin of the shield (F), L3, showing alternating light and dark bands (G). Scale bar equals 3.3 mm in (A), 567 μm in (B), 523 μm in (C), 263 μm in (D) 113 μm in (E), 403 μm in (F) and 279 μm in (G).

The cephalothoracic shield of *Phialaspis* is unusual compared to other heterostracan taxa in that it exhibits considerable histological variation across its width (Fig. 5A). The central region consists of a continuous smooth superficial layer, supplied by a compact stratum of canals (L1), which open into a cancellar layer (L2). The peripheral region is subdivided into a number of regular units defined by conspicuous lines of arrested growth. Each unit consists of a discontinuous superficial layer of crenulated and smooth tubercle ridges, which overlay a discontinuous L1 and L2. L3 forms a continuous basal stratum across the entire shield.

##### Superficial layer

The central region (Fig. 5B) attains a thickness of approximately 250 μm. It is composed of centripetal dentine, which develops about ascending pulp canals measuring approximately 10–30 μm in diameter. Odontoblast canaliculi, measuring 5 μm in diameter, radiate from these vascular spaces and penetrate the dentine layer. The tubercles of the peripheral region (Fig. 5C) measured up to 400–600 μm in height. Each tubercle contains one or two ascending pulp canals, measuring 10–50 μm, from which dentine canaliculi radiate. More elaborate superficial sculpture is produced via superposition of successive tubercle generations. Following superposition, the pulp canals of the primary tubercle are partially or completely in filled by secondary centripetal dentine. There is no evidence of a hypermineralised SCE cap in either the central or peripheral regions of the shield. However, it is worth noting that the terminal rami of the dentine canaliculi frequently perforate the surface of the superficial layer. Furthermore, the crowns of the tubercles are typically irregular, suggesting the superficial layer has experienced at least some degree of wear.

##### Layer 1

The ascending pulp canals of the superficial layer are supplied by a network of compact canals defined by centripetal osteon‐like lamellar tissue, which is largely devoid of unmineralised spaces (Fig. 5B–D). The lamellae of L1 are frequently truncated by vascular spaces (Fig. 5D), suggesting this portion of the shield was remodelled during development. In the central region, L1 forms a continuous stratum underlying the smooth superficial layer (Fig. 5B). In the peripheral region, L2 is discontinuous, occurring as a discrete compact region at the base of each tubercle (Fig. 5C).

##### Layer 2

Variable in thickness. In the central portion, it measured up to 1.4 mm, while in the peripheral region measured up to 800 μm. The central region contains a vascular network comprising large polygonal cancellae and smaller trabecular osteons that interconnect with the canals of L1 (Fig. 5B). The peripheral region contains a vascular network of canals and cancellar chambers (Fig. 5C). The intersecting radial walls, which define the vascular spaces of L2, share similar histology. The core of the intersecting walls is faintly lamellar, or in some places homogenous, and contains a coarse meshwork of thread‐like spaces, (each of which measured approximately 5 μm in diameter) which envelope the vascular spaces. The outer portion of the walls comprises thin centripetal lamellae perforated by an orthogonal fabric of minute thread‐like spaces that deform the lamellar boundaries (Fig. 5E). The fine centripetal lamellae are frequently truncated by vascular spaces, suggesting the vasculature network of L2 was remodelled via resorption (Fig. 5E). Adjacent lamellae show close to the same extinction angle when analysed under polarised light, indicating similar crystal orientations. This is consistent with PFB. Ascending lines of arrested growth, which demarcate previous margins of the shield, are observed in L2 of the peripheral region of the shield (Fig. 5F). These arrested growth lines are overprinted by osteons, suggesting that systematic remodelling via resorption accommodated incremental marginal growth of the shield.

##### Layer 3

Measured up to 450 μm and comprises a substantial proportion of the dermal skeletal thickness. At the centre of the shield, where it reaches its maximum thickness, it is constructed from over 60 ply. If L3 is analysed under cross polarised light, the lamellae appear as alternating light and dark bands, indicting that the crystal orientations of successive lamellae are perpendicular (Fig. 5G). This mineralisation configuration is compatible with Isopedin *sensu* Francillon‐Vieillot et al. (1990). At the margin of the shield, L3 contains a fabric of coarse, orthogonally aligned thread‐like spaces measuring around 3–6 μm in diameter. These are comparable with Sharpey's fibres. The margin of the shield is characterised by ascending lamellae, which are continuous with the ply of L3. L3 is thickened during ontogeny via basal apposition of lamellae.

### Cyathaspidiformes Kiaer, 1930

#### Amphiaspis sp

**Figure 6:**
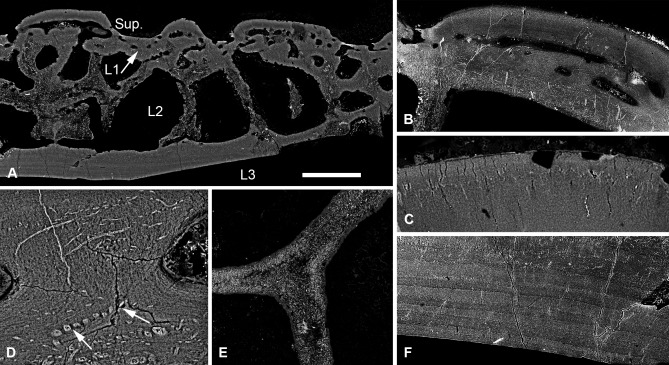
Scanning electron microscopy BSE sections of *Amphiaspis* sp., GIT 313‐32. Architecture of the cephalothoracic shield (**A**); section through a tubercle, showing centripetal dentine and compact vascular spaces of L1 (**B**); detail of the same tubercle, showing canaliculi perforating the surface (**C**); detail of centripetal lamellae which circumscribe the vasculature of L1, arrows points to truncated fibre spaces, interpreted as evidence of resorption (**D**); radial walls of L2, showing a homogenous core (**E**); detail of L3 (**F**). Scale bar equals 0.9 mm in (A), 393 μm in (B), 75 μm in (C), 115 μm in (D), 351 μm in (E) and 182 μm in (F).

The cephalothoracic shield of *Amphiaspis* is four‐layered (Fig. 6A). It consists of a superficial layer of broad round tubercles, a compact layer of canals (L1), a cancellous middle layer (L2) and a continuous lamellar basal layer (L3).

##### Superficial layer

Tubercles measured around 250 μm in height (Fig. 6B). Each tubercle contains a central canal, around 40–60 μm in diameter, about which dentine develops centripetally. Highly polarised odontoblast canulicululae, measuring 1–2 μm in diameter, radiate from the pulp canals and ramify approximately 30 μm from the surface of the tubercles (Fig. 6C). The tubercles appear to lack a capping layer of SCE.

##### Layer 1

Around 200–500 μm thick. Comprises an anastomosing network of vascular canals, defined by centripetal lamellar walls. The lamellar tissue is pervaded by a fine centripetal fabric of thread‐like spaces, each measuring approximately 1–5 μm in diameter. These spaces are concordant with the lamellae and envelope the vascular canals. The centripetal lamellae are frequently truncated by the vascular spaces, indicating the superficial vascular network was remodelled via resorption (Fig. 6D). Below the compact vasculature there is an orthogonal fabric of coarse thread‐like spaces, measuring 8–13 μm in diameter, which pervade the upper part of L2. These are compatible with extrinsic collagen fibre spaces. These fibre spaces terminate abruptly at the boundary with the compact vasculature, supporting the hypothesis that L1 was remodelled during ontogeny (Fig. 6D).

##### Layer 2

Approximately 1.6 mm in thickness. It comprises a network of polygonal cancellae subdivided by robust intersecting radial walls that measured 170–250 μm in section. The radial walls have recrystallised during diagenesis, with the effect that some fine structures, such as lamellar boundaries, have been destroyed. Scanning electron microscopy BSE analysis shows that the core of the walls is chemically distinct from the outer parts, suggesting that these bands represent discrete growth stages. This implies the radial walls developed centripetally like osteons, similar to other heterostracan taxa. Recrystallisation of the intersecting radial walls has accentuated an orthogonal fabric of thread‐like spaces, each measuring approximately 2–5 μm in diameter and spanning the entire width of the walls (Fig. 6E). The walls are pervaded by small holes, measuring 2–10 μm, which are elliptical‐circular in section. It is unclear whether these are original structures or a product of recrystallisation.

##### Layer 3

Measured approximately 400 μm thick and consists of around 11 ply. It is avascular, save for an occasional ascending canal, measuring approximately 150 μm in diameter. Alternate lamellae contain an orthogonal fabric of extremely fine thread‐like spaces, approximately 1 μm in diameter. These terminate abruptly at lamellar boundaries and are consistent with intrinsic collagen fibrils. L3 appears to lack Sharpey's fibres.

#### Anglaspis macculloughi

**Figure 7:**
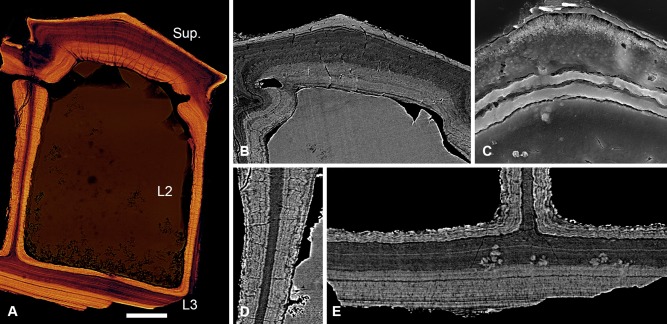
Synchrotron radiation X‐ray tomographic microscopy sections, NHM P.73620 (**A, B, D, E**) and etched SEM sections, NHM P.73621 (**C**) of the cephalothoracic shield of *Anglaspis macculloughi*. Volume rendered virtual thin section, showing the histological structure of the shield (A); section through the superficial layer, showing centripetal dentine capped with a layer of SCE. Canaliculi pervade the dentine and ramify at the enameloid/dentine junction (B); detail of the enameloid capping layer (C); section through a radial wall of L2, showing centripetal lamellae developed about a compact lamellar core. The lamellae are warped in association with an orthogonal fabric of thread‐like spaces (D); section through L3 and the base of L2 (E). Scale bar equals 82 μm (A), 60 μm (B), 12 μm (C) and 41 μm (D, E).

Cyathaspids are characterised by possessing cephalothoracic armour comprising a fused dorsal shield, branchial plates and a ventral disc. The dermal skeleton of *Anglaspis* is three‐layered (Fig. 7A). It consists of a superficial layer of pitched ridges, a cancellous middle layer of large polygonal chambers (L2) and a thin lamellar basal layer (L3). The composite layers of the dermal skeleton are continuous across the cephalothoracic shield.

##### Superficial layer

Approximately 100 μm thick and composed of broad, pitched, elongate ridges, some of which span the entire rostrocaudal extent of the head shield. The ridges are predominantly composed of dentine and capped by a continuous layer of enameloid, up to 20 μm thick (Fig. 7B,C). Much as in other heterostracans, the dentine component of the ridges develops centripetally about a vascular space. Unlike other heterostracans, however, the superficial vasculature forms a continuous space with the underlying polygonal cancellae of the middle layer. The superficial layer is penetrated by highly polarised odontoblast canaliculi, approximately 1–2 μm in diameter. These radiate directly from the underlying polygonal spaces, and also from small canals, around 16 μm in diameter, which link the polygonal spaces to parallel grooves enclosed between adjacent ridges. Odontoblast canaliculi bifurcate at the enameloid/dentine junction and their terminal processes pass into the enameloid layer (Fig. 7B).

##### Layer 2

Constructed from a network of intersecting radial walls defining polygonal chambers, which are interconnected via small perforating canals. The polygonal chambers can span the entire thickness of L2, however, in larger shields they are typically subdivided by cross walls. As in other heterostracans, these walls are constructed of centripetal concentric lamellae (Fig. 7D). The interior‐most lamellae, which form the centre of the vertical walls, are composed of a regular compact tissue, devoid of fibre spaces. This is interpreted as an early mineralisation phase. The base of L2 is composed of similar compact tissue, yet it contains a coarse irregular mesh of loosely packed fibres, each approximately 2–5 μm in diameter, which converge at junctions with the base of the vertical walls. A late mineralisation phase develops centripetally about the cancellae. This tissue is less compact and is penetrated by a fine fabric of thread‐like spaces, less than 1 μm in diameter, which radiate from the older compact lamellar tissue, towards the centre of the polygonal cavities. The lamellar boundaries of this late mineralisation phase appear warped in association with the orthogonal thread‐like spaces (Fig. 7D). The concentric lamellae of the vascular spaces underlying the superficial layer are continuous with the dentine lamellae of the tubercle ridges. The lamellae are centripetally deposited, indicating synchronous, osteon‐like development of the superficial layer and L2. The *Anglaspis* dermal skeleton exhibits no evidence of resorption. Consequently, we can infer that the gross architecture of the *Anglaspis* dermal skeleton was established early during mineralisation.

##### Layer 3

Composed of plywood‐like compact lamellar tissue. It measured up to 55 μm and is constructed from approximately 12–20 ply. Each ply contains a fine fabric of parallel intrinsic fibres, which is aligned orthogonal to the intrinsic fabric of the adjacent ply. Similar to *Amphiaspis*, Sharpey's fibres are conspicuously absent from L3 of *Anglaspis*. The lamellar tissue is penetrated periodically by ascending canals, which pass into the polygonal chambers of the linked vascular system.

### Generalised Demoskeletal Histology of Cyathaspidiformes

Dermal skeleton comprises four layers. The superficial layer is composed of tubercles or ridges, each containing a network of canals or a single continuous vascular space, about which dentine develops centripetally. Polarised ondontoblast canaliculi radiate from vascular spaces and ramify at, or close to, the surface of the tubercle. L1 is formed from an anastomosing network of canals, which supply the pulp canals and interconnect with the cancellae of L2. This layer is conspicuously absent in *Anglaspis*. L2 comprises cancellae defined by an intersecting network of radial walls. These are constructed from a compact core, surrounded by lamellar tissue that develops via centripetal apposition. An orthogonal fabric of thread‐like spaces pervades the walls. These spaces appear to radiate from the core of the walls and noticeably warp the centripetal lamellar boundaries. L3 is composed of isopedin. It is comparatively thin and conspicuously lacks Sharpey's fibres.

### Pteraspidiformes Berg, 1940

#### Pteraspis sp

**Figure 8:**
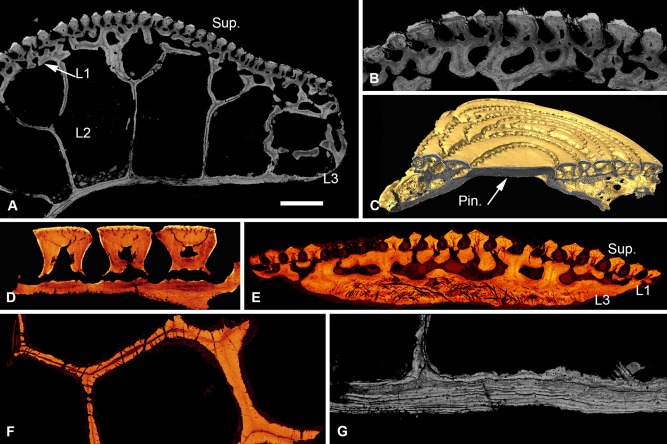
Scanning electron microscopy BSE (**A, B, G**) and SRXTM (**C–F**) histological sections of *Pteraspis* sp. NRM‐PAL C.5943, structure of the cephalothoracic shield of *Pteraspis* (A); detail of the superficial layer and L1 of the same specimen (B); NRM‐PAL C.5944, section through the pineal plate (C); volume rendered virtual thin section of the tubercles, showing the canaliculi radiating from central pulp canals and ramifying at the enameloid/dentine junction, specimen lost during sampling (D); NRM‐PAL C.5945, volume rendered virtual thin section showing histological structure of the body scales (E); transverse volume rendered virtual thin section through L2, showing thread‐like spaces pervading the entire thickness of the radial walls, specimen lost during sampling (F); NRM‐PAL C.5943, section through L3 and the base of L2 (G). Pin., pineal window. Scale bar equals 373 μm in (A), 177 μm in (B), 339 μm in (C), 61 μm in (D), 149 μm in (E), 86 μm in (F) and 123 μm in (G).

The dermal skeleton of *Pteraspis* is four layered (Fig. 8A). It comprises a superficial layer of crenulated ridges, a compact layer of canals (L1), a cancellous layer of polygonal spaces (L2) and a thin lamellar basal layer (L3). The scales of the body are three‐layered and lack L2.

##### Superficial layer

Approximately 80–140 μm thick. It is composed of crenulated ridges of dentine, which are capped with a layer of enameloid up to 15 μm thick (Fig. 8B). The lateral margins of the ridges are serrated, interlocking with those of adjacent ridges like the teeth of a zip (Fig. 8C). This completely encloses the grooves separating adjacent ridges. Each superficial ridge contains a series of ascending pulp canals, around which dentine develops centripetally. Polarised odontoblast canaliculi, approximately 1–2 μm in diameter, radiate from these canals and permeate the dentine. The canaliculi ramify in close proximity to, and either side of, the enameloid/dentine junction (Fig. 8D). The ascending pulp canals are fed via series of longitudinal vascular canals, which run beneath the tubercle ridges. Small perpendicular canals, around 13 μm in diameter, branch at regular intervals from either side of the longitudinal canals, connecting to adjacent separating grooves (Fig. 8D,E). The walls of this pulp network are composed of lamellar centripetal tissue that shows osteon‐like development. The transparent covering of the pineal organ is composed of a single dentine tubercle capped by a continuous layer of SCE. The deeper layers of the dermal skeleton are not present beneath the pineal window, but expand in the surrounding pineal plate (Fig. 8C).

##### Layer 1

Discontinuous across the shield. It measured up to 100–200 μm in thickness and comprises an anastomosing network of vascular canals, defined by radial walls, which interconnect with the superficial pulp network. The canal walls are composed of lamellar centripetal tissue, which shows osteon‐like development. The lamellar tissue is pervaded by occasional thread‐like spaces, however the core of the walls is typically homogenous (Fig. 8B). In the cephalothoracic dermal skeleton, the canals open directly into L2. However, L2 is absent in the scales of the body. Instead, L1 constitutes a continuous network of canals, linking the pulp network of the scale. The vasculature of the scale is supplied by occasional ascending canals perforating L3, which open directly into L1.

##### Layer 2

Measured approximately 1.5 mm in thickness and is continuous across the shield. It comprises a network of polygonal chambers defined by intersecting radial walls and interconnected via numerous small canals. The mineral matrix of the walls is composed of a homogenous core enveloped by thin lamellae showing osteon‐like centripetal development. These tissue types are interpreted as distinct phases of mineralisation. The intersecting walls, which define the vascular network, are perforated by an orthogonal fabric of minute thread‐like spaces, each measuring around 1–2 μm in diameter (Fig. 8F). Some polygonal chambers are partially subdivided by incomplete trabeculae, indicating L2 was remodelled by cross‐wall formation.

##### Layer 3

Extremely thin (approximately 100 μm) and composed of around 12 ply (Fig. 8G). It contains mesh of coarse fibres, each approximately 3–5 μm in diameter, which are oblique relative to the lamellae and aligned in two principal orientations perpendicular to one another (Fig. 8E). These conspicuous fibres penetrate L3 and are consequently interpreted as Sharpey's fibres.

#### Loricopteraspis dairydinglensis

**Figure 9:**
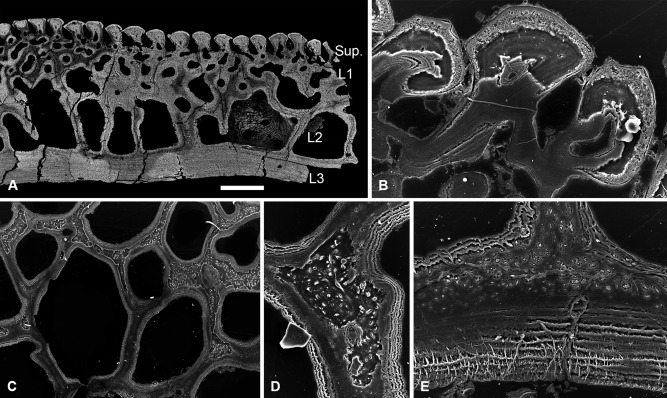
Scanning electron microscopy BSE (NHM P.73622, **A**) and etched SEM (NHM P.73623, **B–E**) sections through the cephalothoracic shield of *Loricopteraspis dairydinglensis*. Architecture of the cephalothoracic dermal skeleton (A); detail of the superficial tubercles, showing centripetal dentine developed about a central pulp canal with a capping layer of SCE (B); detail of L2, showing an intersecting network of radial walls composed of centripetal lamellae with a homogenous core (C); section through a radial wall of L2, showing the homogenous core is pervaded by a mesh of coarse unmineralised spaces. The outer lamellar part is perforated by an orthogonal fabric of fine, thread‐like spaces, which warp the lamellae (D); section through L3, showing an orthogonal fabric of Sharpey's fibres (E). Scale bar equals 319 μm in (A), 50 μm in (B), 233 μm in (C), 71 μm in (D) and 51 μm in (E).

The dermal skeleton of *Loricopteraspis* measured approximately 1.4 mm in thickness and consists of four layers (Fig. 9A): a superficial layer of tubercle ridges, a compact layer of vascular canals (L1), a honeycomb‐like layer of cancellae (L2) and a plywood‐like basal layer (L3).

##### Superficial layer

Approximately 200 μm thick. It consists of parallel tubercle ridges separated by grooves, which appear tear drop‐shaped in section. The tubercles are composed primarily of dentine and are capped by a discontinuous layer of SCE up to 30 μm thick. Each tubercle contains numerous regularly spaced ascending pulp canals, about which dentine develops centripetally and from which dentine canaliculi (measuring up to 5 μm in diameter) radiate (Fig. 9B). The canaliculi penetrate the dentine and the terminal branches permeate the enameloid/dentine boundary. As in ‘*Pteraspis’*, the ascending pulp canals branch from a series of longitudinal canals underlying the tubercles. These are interconnected with the grooves between tubercle ridges via small, regularly spaced canals approximately 20–50 μm in diameter.

##### Layer 1

Measured approximately 200 μm in thickness. It comprises an anastomosing network of vascular canals, each approximately 10–20 μm in diameter, that link the pulp network of the superficial layer with the underlying cancellae. The canals are circumscribed by an intersecting network of radial walls, which show osteon‐like centripetal apposition of lamellae. The lamellar tissue is pervaded by a fine fabric of orthogonal, thread‐like spaces, each measuring approximately 1–2 μm, which appear to warp the lamellae. The core of the walls appears homogenous, and is perforated by a meshwork of thread‐like spaces that envelope the vascular canals.

##### Layer 2

Measured around 850 μm in thickness. The cancellae are linked to each other via small canals measuring approximately 20–100 μm in diameter. The intersecting radial walls, which define the polygonal chambers of L2, are histologically comparable to L1. They are composed of lamellar tissue, which develops via osteon‐like centripetal apposition (Fig. 9C). An orthogonal fabric of thread‐like spaces, each measuring approximately 1–2 μm in diameter, crosscuts the lamellar tissue. The boundaries between lamellae appear to be warped in association with the orthogonal thread‐like spaces (Fig. 9D). The core of the radial walls is typically homogenous and contains a concentric meshwork of coarse, thread‐like spaces approximately 2–4 μm in diameter (Fig. 9D).

##### Layer 3

Measured around 300 μm in thickness and comprises up to 24 ply. L3 is largely avascular with the exception of occasional ascending canals, that measured approximately 50 μm in thickness, which connect to the cancellar layer. L3 contains an orthogonal fabric of coarse, extrinsic fibres (5–8 μm in diameter; Fig. 9E). These are compatible with Sharpey's fibres.

#### Psammosteus megalopteryx

**Figure 10:**
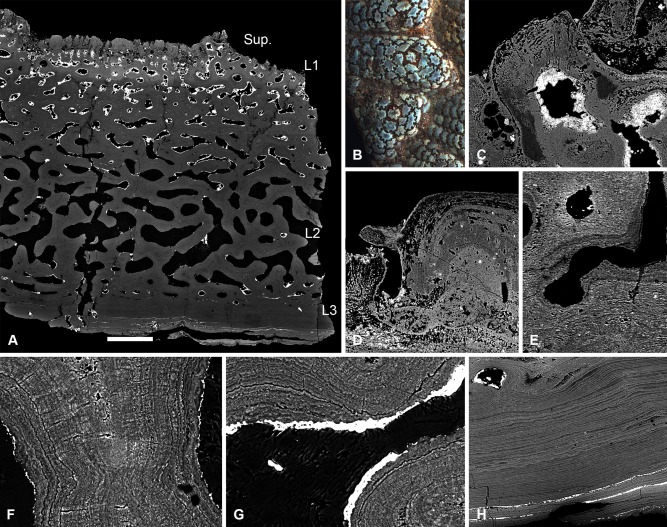
Histology of *Psammosteus megalopteryx*, NHM P.73624. SEM BSE sections (**A, C–H**) and LM image (**B**). Structure of the cephalothoracic dermal skeleton (A), detail of surface ornament consisting of concentric tubercle ‘islands’ separated by grooves (B); section through a tubercle, showing centripetal dentine lamellae pervaded by polarised canaliculi (C); superposition of a second generation of tubercles. The tubercle on the right is completely in filled with secondary dentine (D); section through L1 showing truncation of lamellae by the vasculature, interpreted as evidence of resorption (E); section through a radial wall of L2, showing centripetal apposition of lamellae about a homogenous core. A fine fabric of orthogonal thread‐like spaces passes through and warps the lamellae (F); lamellae truncated by the vascular space, suggesting resorption of L2 (G); detail of L3 (H). Scale bar equals 785 μm in (A), 1.9 mm in (B); 99 μm in (C), 84 μm in (D); 121 μm in (E), 43 μm in (F); 41 μm in (G); 157 μm in (H).

The cephalothoracic dermal skeleton of *Psammosteus* is extremely thick in comparison to other heterostracan taxa (measuring over 5 mm). It comprises a superficial layer of concentric tubercle ‘islands’ supplied by compact vasculature (L1). L2 consists of a continuous trabecular network of vascular canals. L3 is lamellar and well developed (Fig. 10A).

##### Superficial layer

Approximately 250–350 μm thick. Consists of stellate tubercles tightly clustered within discrete concentric ‘islands’, separated by an intersecting network of grooves (Fig. 10B). Each stellate tubercle contains one or several pulp cavities about which dentine develop centripetally (Fig. 10C). Occasionally, the pulp cavities appear to truncate the centripetal lamellae, indicating remodelling of the superficial vascular network. Most of the pulp canals are completely in‐filled by secondary pleromic dentine (Fig. 10D). Polarised dentine canaliculi radiate from the pulp cavities and permeate the dentine. They ramify just below the surface of the tubercle. In contrast to most other heterostracan taxa, the tubercles appear to lack a caping layer of SCE. The superficial layer of *Psammosteus* is composed of multiple tubercle generations (Fig. 10D). Older, heavily abraded tubercles are frequently overlapped or even completely buried beneath second‐generation tubercles. This kind of growth was described as ‘local areo‐superpositional’ by Ørvig (1961, 1978).

##### Layer 1

Measured approximately 250–450 μm in thickness. It comprises discrete regions of compact vasculature, which underlie each concentric tubercle ‘island’. These vascular regions are separated from each other by deep channels, which open externally and perforate L1 (Fig. 10A). The vascular canals of L1 are defined by centripetal lamellar walls, which show osteon‐like development. These centripetal lamellae are sometimes truncated by the vascular spaces (Fig. 10E), suggesting this layer was remodelled via resorption. The lamellae are pervaded by an orthogonal fabric of thread‐like spaces, identical to those present in L2. SEM BSE analysis reveals that L1 is chemically distinct from L2. A discontinuity exists between these layers, which is interpreted as a line of arrested growth demarking two distinct phases of mineralisation (Fig. 10A).

##### Layer 2

Measured approximately 250–540 mm. It is trabecular and contains an anastomosing network of vascular canals that interconnects with L1. The intersecting radial walls, which define the vascular spaces, are composed of a homogenous core about which a fine lamellar centripetal tissue develops, enveloping the vascular spaces in a manner comparable with osteons. The core of the walls is penetrated by a coarse meshwork of thread‐like spaces, each of which measured approximately 1–3 μm in diameter. The concentric lamellar tissue is pervaded by a discrete fabric of extremely regular orthogonal thread‐like spaces, each approximately 1 μm in diameter (Fig. 10F). The boundaries between lamellae appear distorted in association with this fabric. Centripetal lamellae are occasionally truncated by the vasculature, suggesting canal spaces were remodelled during growth via resorption (Fig. 10G).

##### Layer 3

Constitutes a substantial proportion of the dermal skeletal thickness. It is constructed from over 90 ply (c. 500–750 μm thick). It is avascular, save for the occasional ascending canal opening into the vascular network of L2 (Fig. 10H). It is permeated by numerous small thread‐like spaces (each approximately 2 μm in diameter) oriented orthogonally to, and cross cutting, the lamellae. These are compatible with Sharpey's fibres.

### Generalised Dermal Skeletal Histology of Pteraspidiformes

The pteraspid dermal skeleton is composed of four layers (superficial, L1, L2 and L3). The superficial layer is constructed from serrated tubercle ridges separated by flask‐shaped grooves. The tubercles are composed predominantly of dentine, which develops centripetally about ascending pulp canals. A thin layer of SCE caps each tubercle. Odontoblast canaliculi radiate from the pulp canals and pervade the dentine, ramifying at, or close to, the enameloid/dentine junction. The ascending pulp canals are linked via a pulp network comprising a series of longitudinal canals that directly underly the tubercles. These open into the flask‐shaped grooves via smaller, perpendicular canals. The pulp network is intimately interlinked with the compact canals of L1. These open into underlying cancellae of L2, which, in turn, are interconnected to one another via small canals. Both the trabecular and cancellar spaces are defined by an intersecting network of radial walls. These are constructed of lamellar tissue, which develops centripetally like osteons. The lamellar tissue contains an orthogonal fabric of thread‐like spaces, which cross cut the walls. Frequently the core of the walls appears homogenous as a result of remodelling. L3 is comparatively thin and has a plywood‐like structure. It is avascular, with the exception of occasional ascending canals, and contains a coarse fabric of orthogonal Sharpey's fibres aligned either parallel to one another, or inclined in two principal orientations perpendicular to one another.

The psammosteid dermal skeleton deviates significantly from the generalised pteraspid histology. The superficial layer comprises multiple generations of tubercles (rather than tubercle ridges) and that appear to lack a capping layer of SCE. L2 is trabecular and lacks polygonal cancellae. L3 incorporates many ply and is extremely thick. *P. megalopteryx* exhibits typical psammosteid histology, based on comparison to previous studies (Gross, 1930, 1935; Bystrow, 1955; Tarlo and Tarlo, 1961; Halstead Tarlo, 1963, 1964b; Johanson et al., 2013). Given that the position of psammosteids within pteraspidiformes is well supported based on the homology of the dermal skeletal plates (Blieck, 1984; Janvier, 1996; Pernegre and Elliott, 2008), it seems likely that the psammosteid dermal skeleton is highly autapomorphic.

## DISCUSSION

### The Pleisiomorphic Condition of the Heterostracan Dermal Skeleton

**Figure 11:**
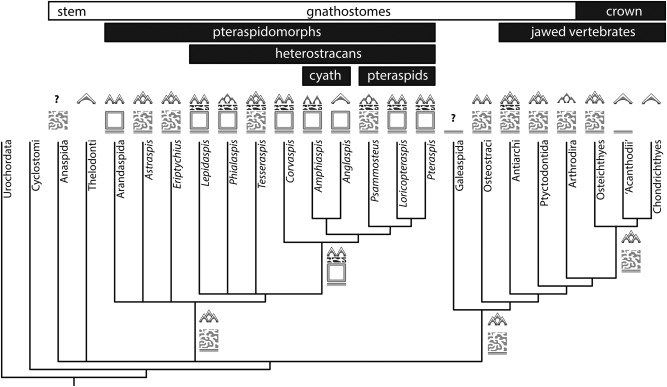
Evolutionary distribution of tissue types among total‐group gnathostomes. Phylogenetic relationships of pteraspidomorphs based on Janvier (1996), relationships among stem‐gnathostomes follow Donoghue and Keating (2014). Jawed vertebrate relations follow Zhu et al. (2013). Symbols represent the four‐layered structure of the dermal skeleton. Grey triangles correspond to dentine tubercles; white caps correspond to a capping layer of enameloid; stacked triangles corresponds to stacked tubercle generations; dark grey reticular band corresponds to L1; square box corresponds to cancellar L2; light grey reticular box corresponds to trabecular L2; bottom‐most band corresponds to L3. Symbols adjacent to internal branches represent the reconstructed ancestral state.

Our survey of the skeletal histology of major heterostracan lineages has revealed substantial variability with respect to both the construction of the dermal skeleton and the microstructure of the component tissues. Nonetheless, our results indicate these disparate skeletal structures are variations on ostensibly the same gestalt. In order to infer the pleisiomorphic nature of the heterostracan dermal skeleton, we must consider this histological variability within a phylogenetic context. Unfortunately, relationships among heterostracans are currently poorly resolved. Here, we adopt the phylogeny from Janvier (1996), which remains the most comprehensive hypothesis of heterostracan relationships (Fig. 11). This tree topology accords with the stratigraphic distribution of heterostracan taxa (Halstead, 1973; Dineley and Loeffler, 1976) and reflects current uncertainty surrounding the placement of tessellate forms. The cyathaspidiforms and pteraspidiforms are regarded as monophyletic sister‐groups, while *Corvaspis* is considered the sister‐taxon to these major clades (based on the list of synapomorphies in Janvier (1996)). The tessellate taxa *Lepidaspis, Tesseraspis* and *Phialaspis* are placed in a polytomy at the base of the Heterostraci.

All of the heterostracan taxa surveyed possess a superficial layer composed of dentine tubercles, which develop centripetally about pulp canals. The dentine is perforated by highly polarised canaliculi, which ramify as they approach the external surface of the tubercle, usually at, or close to, the enameloid/dentine junction. This is consistent with orthodentine *sensu* Smith and Hall (1993). The tubercles are surmounted by a layer of SCE in all taxa except *Phialaspis, Amphiaspis* and *Psammosteus*. Given that enameloid is present in cyathaspids, pteraspids, numerous basal tessellate heterostracans and within the immediate heterostracan outgroups, for example, *Astraspis* (Sansom et al., 1997), we interpret this as a plesiomorphic feature of the heterostracan dermal skeleton. The morphology of the superficial tubercles dictates the architecture of the pulp vasculature. In taxa bearing highly regular tubercle ridges (e.g., cyathaspids and pteraspids), the ascending pulp canals are supplied by a similarly regular vascular network, linked via small canals that open into the grooves between ridges. In contrast, the tubercles of taxa such as *Lepidaspis* and *Corvaspis* contain discrete vasculature, which opens into the deeper layers of the skeleton. This kind of superficial ornament is present in the sister group of pteraspids, the sister group of cyathaspids, the sister group of cyathaspidiformes + pteraspidiformes and in each taxon currently within the basal polytomy of tessellate heterostracans. This implies that tubercle ridges evolved independently in pteraspids and cyathaspids and that separate tubercles epitomises the plesiomorphic condition of the heterostracan superficial layer.

The superficial layer of numerous heterostracan taxa consists of multiple tubercle generations. Some, for example *Psammosteus*, exhibit predominantly successive superposition of tubercles, where as other taxa, such as *Lepidaspis*, exhibit mainly marginal accretion of discrete tubercles. Yet our data suggest the growth of the superficial layer in all heterostracans follows the same underlying pattern. During the first growth episode, primary odontodes develop in association with primary growth of the basal plate. Subsequently, secondary ondontodes are added superpositionally and primary odontodes are added marginally in association with growth of the basal plate. In this manner, the superficial layer can be expanded both marginally and vertically. Variation in growth patterning of the superficial layer can thus be explained by differences in the amount of superposition relative to marginal growth of primary odontodes.

All heterostracan taxa examined, with the exception of *Anglaspis*, possess a compact vascular layer (L1), which supply the pulp canals of the superficial network. L1 consists of an anastomosing network of vascular canals, which are intimately interlinked with the upper division of L2 to the extent that the centripetal lamellae (which circumscribe the canal walls) are continuous with the centripetal tissue of L2. The tissue appears comparable to the acellular PFB that forms the walls of L2, however, the conspicuous fabric of thread‐like spaces is less well developed. Developmental and histological studies of recent gnathostomes suggest that odontodes develop in association with ‘bone of attachment’ (Smith, 1979; Reif, 1982; Smith and Hall, 1990, 1993). We have found no direct evidence of such a tissue in the dermal skeleton of any heterostracan, although it is possible that L1 develops via resorption of the bone of attachment during ontogeny. This notion is supported by evidence of truncated lamellae in several taxa, (see Figs. 6D and 10E), suggesting L1 was remodelled through ontogeny.

L2 is constructed from a network of intersecting radial walls, which circumscribe vascular spaces. The radial walls develop via centripetal apposition, like osteons, forming concentric lamellae. This lamellar fabric is acellular in the sense that it does not contain cell lacunae formed by entrapment of osteoblasts. The tissue contains an orthogonal fabric of thread‐like spaces, which crosscut the lamellae. This is consistent with aspidin, as defined by Gross (1930). Contiguous aspidin lamellae contain a fabric of parallel intrinsic collagen fibrils, indicating aspidin is an acellular type of PFB.

The intersecting radial walls, which comprise L2, circumscribe cancellous or compact vascular spaces. Yet despite the apparent distinctions between these two architectures, there is no phylogenetic consistency in their distribution (see Fig. 11). Furthermore, some taxa, such as *Corvaspis* and *Phialaspis*, exhibit both trabecular and cancellous regions across their shields, indicating distinction between these two architectures is far from fundamental. We have also presented evidence of resorption of L2 within two disparate lineages (*Phialaspis* and *Psammosteus*), which suggests it is a pleisiomorphic heterostracan character. Furthermore, observations of incomplete cross walls within the dermal skeleton of both ‘*Pteraspis*’ and *Lepidaspis* indicate cancellae were subdivided during ontogeny. Taken together, this evidence indicates L2 was capable of radical remodelling during ontogeny via resorption and redeposition. Gross (1930) was therefore, correct in his assertion that heterostracan dermal skeletal growth was facilitated by resorption. Given this new insight into the dynamic growth patterning of the heterostracan skeleton, the architectural variability of L2 can be interpreted as a product of subtle intertaxon differences in either the timing or rate of cross wall formation and/or cross wall destruction via resorption. A compact trabecular L2 is prevalent in larger taxa with thicker shields, such as the psammosteids, *Rhinopteraspis* and *Tesseraspis* (Fahlbusch, 1957; Halstead Tarlo, 1964b), suggesting development of a trabecular L2 may, in fact, be a structural response to increasing the thickness of the dermal skeleton.

All taxa possess an acellular basal layer (L3) consisting of numerous ply, each containing a regular fabric of inclined collagen fibrils aligned orthogonally to those of contiguous ply. This is consistent with isopedin. L3 is perforated by coarse extrinsic fibres, compatible with Sharpey's fibres, which would have anchored the cephalothoracic shield and scales to the dermis in life. Typically, these fibres are aligned parallel to one another and orthogonal to the basal ply, however, in some taxa (*Lepidaspis*, ‘*Pteraspis*’) they are aligned in two principal orientations perpendicular to one another, forming a cross‐woven mesh. L3 is thickened through ontogeny via incremental apposition of ply at the base of the dermal skeleton.

In summary, the plesiomorphic heterostracan dermal skeleton comprises an odontogenic superficial layer, consisting of discrete tubercles composed of orthodentine and surmounted by SCE caps. These are fed by a compact layer of canals (L1), which link the superficial pulp canals with the vasculature of the middle layer (L2). L2 comprises compact or cancellous layer of acellular PFB (aspidin), which develops via osteon‐like centripetal apposition. The base of the dermal skeleton (L3) consists of a plywood‐like layer of acellular isopedin, which is thickened incrementally via basal apposition. Growth of the shield was achieved via marginal accretion of tubercles and the associated dermal skeleton.

### The Pleisiomorphic Condition of the Pteraspidomorph Dermal Skeleton

Phylogenetic analyses consistently recover the Heterostraci, together with the Ordovician Arandaspida, *Eriptychius* and *Astraspis*, within the clade Pteraspidomorphi (Heterostracomorphi). However, conflicting topologies (e.g., Donoghue et al., 2000; Donoghue and Smith, 2001; Sansom et al., 2010) betray the uncertainty in discerning the relationships between these taxa. Arandaspids, *Eriptychius* and *Astraspis* have classically been interpreted as possessing a three‐layered skeleton, consisting of a superficial layer of dentine tubercles surmounted by enameloid caps, a vascularised middle layer (equivalent to L2) and a lamellar basal layer (equivalent to L3) (Denison, 1967; Sansom et al., 2005). Yet considerable variability exists between these taxa with respect to both the gross architecture of the dermal skeleton and the microstructure of the mineralised tissues. In *Eriptychius* and *Astraspis*, the superficial layer consists of stacked or overlapping generations of orthodentine tubercles, each with an enameloid capping layer, while L2 is trabecular and constructed from acellular lamellar osteons resembling aspidin (Denison, 1967; Ossian and Halseth, 1976; Sansom et al., 1997). In arandaspids, the superficial layer consists of a single stratum of tubercle ridges, while L2 is cancellar (Gagnier, 1993; Sansom et al., 2005; Sansom et al., 2013). Sansom et al. (2013) have also demonstrated that the arandaspid dermal skeleton is composed of cellular tissues rather than orthodentine and aspidin. Given that cellular tissues occur in just this single lineage of pteraspidomorphs, we can infer that the arandaspid dermal skeleton is autapomorphic with respect to the other lineages. Additionally, the inferred plesiomorphic heterostracan dermal skeleton is consistent with that of *Eriptychius* and *Astraspis*. Thus, we conclude that the dermal skeleton of ancestral pteraspidomorphs was three‐layered, consisting of a stack of superficial tubercles composed of orthodentine and enameloid, a trabecular middle layer of aspidin (L2) and a basal lamellar layer (L3).

### The Pleisiomorphic Condition of the Vertebrate Dermal Skeleton

In order to infer the plesiomorphic histology of the vertebrate dermal skeleton, it is necessary to draw homology between the dermal skeletons of disparate ostracoderm lineages. However, each lineage is distinct in terms of its dermal skeletal architecture and tissue microstructure, thus inferring homology between these disparate skeletons is by no means trivial. Four criteria are informative in drawing tissue homologies: a) microstructure (i.e., whether tissues are structurally comparable), b) tissue skeletogenesis (i.e., whether tissues are developmentally comparable), c) topology (i.e., whether tissues occur in equivalent layers of the dermal skeleton) and d) phylogenetic distribution (i.e., whether the distribution of tissues within a tree supports a hypothesis of homology). All criteria must be met if a statement of homology is to be accepted. For example, while conodont crown tissue is structurally, skeletogenically and topologically comparable to enameloid, Murdock et al. (2013) have shown that this hypothesis of homology fails the test of phylogenetic congruence.

The dermal skeleton of osteostracans comprises a superficial layer of dentine tubercles surmounted by enameloid caps, a vascular middle layer (equivalent to L2) constructed of true osteons (with osteocyte lacunae) and a lamellar basal layer (equivalent to L3) comparable to isopedin (Gross, 1935, 1961; Denison, 1951; Smith and Hall, 1990; Donoghue et al., 2006). Giles et al. (2013) demonstrated that this dermal architecture is also plesiomorphic with respect to placoderms, regardless of whether they are perceived as monophyletic or paraphyletic. Osteichthyans possess comparable dermal skeletal architecture, although their tubercles are capped with ganoine or enamel [ganoine is a hypermineralised tissue developmentally comparable to enamel (Sire et al., 1987; Sire, 1994; Sire et al., 2009)]. Chondrichthyans, the sister group to all other crown‐gnathostomes, possess dermal denticles capped with enameloid, thus the phylogenetic distribution of dermal skeletal capping tissues suggests ganoine is apomorphic with respect to osteichthyans.

Chondrichthyan dermal denticles (placoid scales) equate to the superficial layer of the tripartite dermal skeleton. While a shark‐like micromeric dermal skeleton, composed of placoid‐type scales, has been traditionally interpreted to represent a primitive condition (Hertwig, 1874, 1876, 1879; Klaatsch, 1890; Goodrich, 1907; Ørvig, 1951; Stensiö, 1958, 1961, 1962, 1964; Reif, 1973, 1976, 1982), a phylogenetic perspective demonstrates that the deeper layers of the dermal skeleton have been secondarily lost in the chondricthyan lineage. This is supported by recent phylogenetic analyses, which place the more extensively skeletonised acanthodians as a paraphyly branching from the chondrichthyans stem‐lineage (Brazeau, 2009; Davis et al., 2012; Zhu et al., 2013). Thus, contrary to the conventional view, it seems that extant sharks provide a poor model for the early vertebrate dermal skeleton.

Galeaspids are consistently resolved as the sister‐group of osteostracans + jawed vertebrates, yet their dermal histology deviates from the tripartite dermal skeleton exhibited by pteraspidomorphs, osteostracans, placoderms + osteichthyans. In galeaspids, the dermal skeleton is constructed from a superficial layer of tubercles composed of spheritic bone and a basal plate of ‘galeaspidin’ (a type of isopedin characterised by a pervading fabric of mineralised fibre bundles; Wang et al., 2005).

The anaspid dermal skeleton is poorly characterised and even more poorly understood, principally because its histology has been characterised with insufficient detail. Gross (1938, 1954, 1958) described scales from the Devonian of England, Norway and Estonia as consisting entirely of an acellular lamellar tissue, which he identified as aspidin. However, it is unclear whether this lamellar tissue is compatible with the centripetal tissue of L2 in pteraspidomorphs, which, definitively, is comprised of ‘aspidin’. Perhaps closer comparison may be drawn with the isopedin layer (L3) of the pteraspidomorph dermal skeleton. However, unlike isopedin, the anaspid lamellar tissue is often extensively vascularised (Gross, 1958; Blom et al., 2001; Donoghue and Sansom, 2002; Donoghue et al., 2006). Anaspids scales are almost always ornamented with tubercles, yet it is uncertain whether these correspond to odontodes. Gross described the tubercle tissue as possessing a radial fabric similar to the tubercles of *Astraspis*, yet detailed microstructure of this tissue is wanting and, as such, it remains unclear whether anaspid tubercles are composed of dentine or bone. Rudimentary understanding of their histology has impacted our ability to constrain the phylogenetic position of anaspids. As such, they have been considered both as the sister‐group to all other skeletonising vertebrates (Donoghue and Smith, 2001; Gess et al., 2006; Sansom et al., 2010; Turner et al., 2010; Conway Morris and Caron, 2014) and nested within the heterostracan + jawed vertebrate clade (Donoghue et al., 2000).

The thelodont dermal skeleton consists of denticles composed of dentine, enameloid and acellular bone of attachment (Donoghue et al., 2006). These denticles are homologous with the superficial layer of the tripartite skeleton shared by heterostracomophs + jawed vertebrates. Like anaspids, however, the phylogenetic position of thelodonts is poorly resolved and it is currently uncertain whether they fall within the pteraspidomorph + jawed vertebrate clade. What is more, some studies have cast doubt on the monophyletic status of thelodonts (Donoghue and Keating, 2014; Donoghue and Smith, 2001).

Based on our understanding of vertebrate relationships we infer that a tripartite dermal architecture comprising a superficial layer of odontodes composed of dentine and capped by enameloid, a middle layer of osteons (equivalent to L2) and a basal isopedin‐like layer (equivalent to L3) is plesiomorphic with respect to pteraspidomorphs, osteostracans and jawed vertebrates. Galeaspids noticeably deviate from this dermal skeletal architecture, yet their ‘galeaspidin’ layer is structurally, skeletogenically and topologically comparable to the isopedin layer (L3) which must have been present in the last ancestor of galeaspids + pteraspidomorphs. It, therefore, seems likely that the galeaspid basal plate is a derivative of L3. Rudimentary understanding of the histology and phylogenetic position of anaspids currently prohibits insight into the plesiomorphic vertebrate skeleton. Nevertheless, an extensive osteogenic component of the dermal skeleton is present in the sister group of all other skeletonising vertebrates, regardless of competing tree topologies (Donoghue et al., 2000; Gess et al., 2006; Sansom et al., 2010; Turner et al., 2010; Conway Morris and Caron, 2014). Thus, while we cannot currently resolve whether a tripartite skeleton was present in the ancestor of skeletonising vertebrates, we can interpret the absence of an osteogeneic dermal skeletal component in thelodonts as a derived condition. Greater understanding of anaspid histology will no doubt elucidate the ancestral condition of dermal bone and clarify the evolutionary acquisition of a stratified tripartite dermal skeleton.

## CONCLUSIONS

We have surveyed the histology of dermal skeletons spanning heterostracan diversity. Our results reveal the plesiomorphic heterostracan skeleton is four‐layered. The superficial layer consists of odontodes composed of dentine and SCE, which are added, both superpositionally and marginally, in association with growth of the underlying dermal skeleton. The odontodes are superimposed upon a compact layer of vascular canals (L1), which supply the pulp canals. The middle layer (L2) is trabecular, and consists of vascular canals circumscribed by centripetal acellular PFB (aspidin) exhibiting osteon‐like development. This layer is remodelled through ontogeny via resorption and redeposition in order to accommodate growth. The basal layer (L3) consists of acellular, plywood‐like, lamellar tissue compatible with isopedin, which thickens through ontogeny via basal apposition of ply. A three layered dermal skeleton, equivalent to the superficial layer, L2 and L3 of the heterostracan skeleton, is plesiomorphic for the pteraspidomorph + gnathostome clade and lost independently in galeaspids and chondrichthyans. The histology and phylogenetic affinity of thelodonts and anaspids is currently poorly understood. Nonetheless, regardless of competing phylogenetic hypotheses, it is clear that the ancestral vertebrate skeleton is composed, at least in part, of osteogenic tissues. Consequently, we can conclude that an exclusively odontogenic, placoid‐type dermal skeleton, such as is present in thelodonts, is unrepresentative of the ancestral vertebrate skeleton.

## ACKNOWLEDGMENTS

The authors would like to thank Stuart Kearns (University of Bristol) for his assistance using the SEM facility at the School of Earth Sciences, University of Bristol as well as Martin Rücklin (NBC, Leiden), John Cunningham, (NRM, Stockholm), Stefan Bengtson (NRM Stockholm), Duncan Murdock (University of Leicester), Carlos Martinez‐Perez (University of Bristol), Federica Marone (PSI) and Marco Stampanoni (PSI) for help at the beamline, David Button (University of Bristol) for help using Aviso and Christopher Rogers (University of Bristol) for assistance with LM photography. We thank Philippe Janvier for his comments, which no doubt improved the quality of this manuscript, Georgy Koentges (Warwick, UK) and Zerina Johanson (NHM UK) for discussion, Emma Bernard (NHM, UK) and Elga Mark‐Kurik (Institute of Geology, Tallinn), for providing access to some of the materials on which this study was based. The contribution from CLM was undertaken in partial fulfilment of an MSc in Palaeobiology at the University of Bristol.
